# Genetics and Epigenetics of Human Pubertal Timing: The Contribution of Genes Associated With Central Precocious Puberty

**DOI:** 10.1210/jendso/bvae228

**Published:** 2025-01-21

**Authors:** Ana Pinheiro Machado Canton, Delanie Bulcao Macedo, Ana Paula Abreu, Ana Claudia Latronico

**Affiliations:** Cellular and Molecular Endocrinology Laboratory LIM/25, Division of Endocrinology and Metabolism, Clinicas Hospital, School of Medicine, University of Sao Paulo, 01246-903 Sao Paulo, Brazil; Integrated Medical Care Center, Center for Health Sciences, University of Fortaleza (Unifor), Fortaleza 60811-905, Brazil; Division of Endocrinology, Diabetes and Hypertension, Brigham and Women's Hospital, Harvard Medical School, Boston, MA 02115, USA; Cellular and Molecular Endocrinology Laboratory LIM/25, Division of Endocrinology and Metabolism, Clinicas Hospital, School of Medicine, University of Sao Paulo, 01246-903 Sao Paulo, Brazil; Discipline of Endocrinology and Metabolism, Department of Internal Medicine, School of Medicine, University of Sao Paulo, 05403-000, Sao Paulo, Brazil

**Keywords:** central precocious puberty, epigenetics, genetics, *MKRN3*, *DLK1*, *MECP2*

## Abstract

Human puberty is a dynamic biological process determined by the increase in the pulsatile secretion of GnRH triggered by distinct factors not fully understood. Current knowledge reveals fine tuning between an increase in stimulatory factors and a decrease in inhibitory factors, where genetic and epigenetic factors have been indicated as key players in the regulation of puberty onset by distinct lines of evidence. Central precocious puberty (CPP) results from the premature reactivation of pulsatile secretion of GnRH. In the past decade, the identification of genetic causes of CPP has largely expanded, revealing hypothalamic regulatory factors of pubertal timing. Among them, 3 genes associated with CPP are linked to mechanisms involving DNA methylation, reinforcing the strong role of epigenetics underlying this disorder. Loss-of-function mutations in Makorin Ring-Finger Protein 3 (*MKRN3*) and Delta-Like Non-Canonical Notch Ligand 1 (*DLK1*), 2 autosomal maternally imprinted genes, have been described as relevant monogenic causes of CPP with the phenotype exclusively associated with paternal transmission. *MKRN3* has proven to be a key component of the hypothalamic inhibitory input on GnRH neurons through different mechanisms. Additionally, rare heterozygous variants in the Methyl-CpG-Binding Protein 2 (*MECP2*), an X-linked gene that is a key factor of DNA methylation machinery, were identified in girls with sporadic CPP with or without neurodevelopmental disorders. In this mini-review, we focus on how the identification of genetic causes of CPP has revealed epigenetic regulators of human pubertal timing, summarizing the latest knowledge on the associations of puberty with *MKRN3*, *DLK1*, and *MECP2*.

Human puberty is a dynamic biological process marked by the appearance of secondary sexual characteristics, acceleration of linear growth, and gonadal maturation, resulting in the acquisition of reproductive capacity [1]. Reproduction is controlled by the hypothalamic-pituitary-gonadal (HPG) axis, a highly hierarchical neuroendocrine axis, whose upper component is GnRH, a hypothalamic factor secreted in a pulsatile manner [2]. The onset of puberty is determined by the increase in the amplitude and frequency of GnRH pulses in the hypothalamus [3]. It is well-established that this pulsatile GnRH secretion is essential for activating and maintaining the HPG axis. But what regulates the reactivation of pulsatile GnRH secretion? What are the trigger factors of puberty? In fact, the identification of regulators of pubertal timing has been a major focus of research in science and medicine [4]. In the past 2 decades, basic and clinical researchers have largely contributed to address these questions, but the completeness of puberty control remains to be fully understood.

The current knowledge on regulators of pubertal timing has revealed that there is fine tuning between an increase in stimulatory factors and a decrease in inhibitory factors [2, 5]. Importantly, among these factors, kisspeptin (encoded by the *KISS1* gene) has a fundamental role as a very potent stimulatory factor required for GnRH secretion. In mammals, 2 main hypothalamic neuronal populations express kisspeptin: 1 at the anteroventral periventricular nucleus (AVPV) and the other at the arcuate nucleus (ARC) [2, 3]. A group of kisspeptin neurons in the ARC coexpress neurokinin B (encoded by the *TAC3* gene; a kisspeptin stimulator) and dynorphin (a kisspeptin inhibitor), which are called KNDy neurons. KNDy neurons have been recognized as key elements in the modulation of pulsatile GnRH secretion and pubertal timing [2, 3]. Notably, strong evidence has confirmed the crucial role of genetic factors in the control of pubertal timing. Valuable contributions have come from experimental studies, including animal models, and from genome-wide association studies (GWAS), looking for common variants associated with small effects on pubertal timing in large populations [4, 6]. Besides them, another successful strategy to identify these regulators of puberty has been also the study of individuals with rare pubertal disorders because it may contribute to elucidating reproductive biology in addition to ultimately providing clinical benefits [6-8]. The growing advances in high-throughput genetic technologies has enabled the identification of multiple genetic factors associated with human pubertal disorders, revealing genes related with the central neuroendocrine control of the HPG axis, especially with the GnRH network.

GnRH-dependent pubertal disorders may have opposite clinical manifestations, depending on the underlying mechanisms. The deficiency of production, secretion, or action of GnRH leads to delayed or absent puberty [6]. Conversely, premature reactivation of pulsatile secretion of GnRH leads to gonadotropin-dependent precocious puberty, also known as central precocious puberty (CPP) [3]. In clinical practice, precocious puberty is defined as the development of secondary sexual characteristics before the age of 8 years in girls and 9 years in boys [1, 3]. The most common form of precocious puberty is CPP, a hypothalamic disorder that mimics physiological puberty but occurs at an inappropriate age. Regardless of cohort, the frequency of CPP has been reported as markedly higher in females, in a proportion about 10 to 20 times higher than in males [9].

Remarkably, an intriguing aspect from genetic studies performed in patients with CPP has been the identification of genetic conditions involving factors somehow linked to mechanisms involving DNA methylation, a major epigenetic mechanism (Table 1 and Fig. 1). In fact, compelling evidence has indicated the participation of epigenetic factors behind the scenes of pubertal timing regulation [10]. There is an emerging concept that a switch from epigenetic repression to activation is a core mechanism underlying the initiation of puberty [11]. Notably, very elegant experimental studies have shown the participation of distinct epigenetic regulatory mechanisms in kisspeptin and GnRH neurons. Briefly, 2 major families of proteins, composed of transcriptional regulators driven by epigenetic mechanisms, were identified with a reciprocal activity of repressive or stimulating function over the kisspeptin system; the Polycomb group and the Trithorax group, respectively [11, 12]. Animal studies have also suggested zinc finger protein-mediated transcriptional repression and microRNA switch regulation as additional epigenetic mechanisms modulating puberty [13, 14]. The review of these experimental studies was previously well and comprehensively addressed and is available elsewhere [2, 11]. Here, we focus on findings from human studies in patients with CPP and summarize how the identification of genetic causes of CPP has revealed genetic and epigenetic mechanisms underlying the control of pubertal timing.

**Figure 1.**
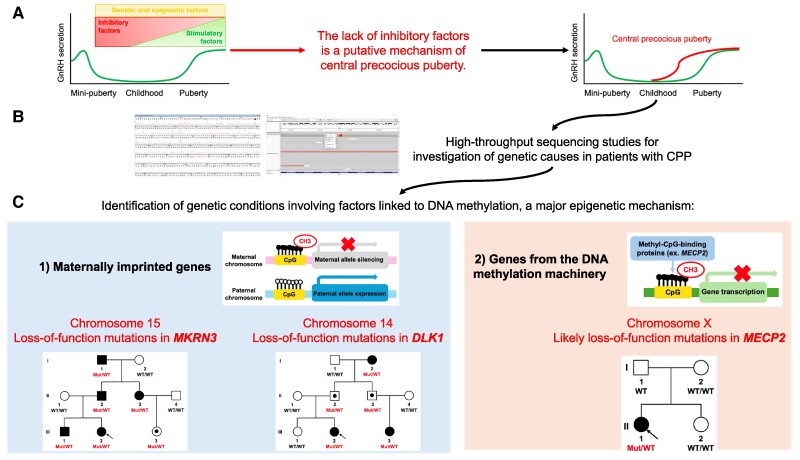
The discovery of genetic causes of central precocious puberty and the revelation of epigenetics behind the scenes. (A) Human puberty is determined by the increase in the pulsatile secretion of GnRH triggered by a dynamic process, involving an increase in stimulatory factors and a decrease in inhibitory factors with strong influence of genetics and epigenetics. Therefore, the lack of inhibitory factors is a putative mechanism for premature reactivation of pulsatile secretion of GnRH, known as central precocious puberty. (B) Innovative high-throughput sequencing studies has been used to investigate genetic causes in patients with CPP. (C) Genetic causes identified in patients with CPP has revealed the loss of function of inhibitory factors linked to mechanisms involving DNA methylation, a major epigenetic mechanism, reinforcing the crucial role of genetics and epigenetics in the regulation of pubertal timing. Two clusters of genetic causes involving epigenetic mechanisms were identified so far: (1) loss-of-function mutations in Makorin Ring-Finger Protein 3 (*MKRN3*) and Delta-Like Non-Canonical Notch Ligand 1 (*DLK1*), 2 autosomal maternally imprinted genes, are relevant monogenic causes of CPP with the phenotype exclusively associated with paternal transmission; (2) likely loss-of-function mutations in Methyl-CpG-Binding Protein 2 (*MECP2*), an X-linked gene that is a key factor of DNA methylation machinery, were recently identified in patients with sporadic CPP with or without neurodevelopmental disorders.

**Table 1.** Monogenic causes associated with central precocious puberty (CPP)

Gene (OMIM)	Locus	Protein function	Inheritance pattern	Most common CPP pattern	Mutation types	Main clinical features
*KISS1R^a^* (604161)	19p13.3	Kisspeptin receptor	Adopted girl	Sporadic	Gain-of-function missense mutation	Very early onset thelarche progressing to CPP Only 1 girl reported
*KISS1^a^* (603286)	1q32.1	Potent GnRH stimulator	Autosomal dominant with incomplete penetrance	Sporadic	Gain-of-function missense mutation	Very early onset CPP Only 1 boy reported
*MKRN3* (603856)	15q11.2	Zinc-finger protein E3 ubiquitin ligase Protein ubiquitination mRNA binding	Autosomal dominant with maternal imprinting	Familial with paternal transmission	Loss-of-function mutations: Missense Frameshift Nonsense Whole-gene deletions Promoter region deletions	Clinically indistinct from idiopathic CPP Mean age at first pubertal signs: Girls: 6.2 (±1.2) years Boys: 7.1 (±1.5) years
*DLK1* (176290)	14q32.2	Noncanonical ligand of Notch pathway Negative regulator of adipogenesis Potential regulator of neurogenesis	Autosomal dominant with maternal imprinting	Familial with paternal transmission	Loss-of-function mutations: Frameshift Nonsense Splice site Intragenic deletions	In adulthood: Overweight/obesity Hyperlipidemia Glucose intolerance/type 2 diabetes
*MECP2* (300005)	Xq28	DNA methylation reader Gene transcription regulator Neurodevelopment factor	X-linked dominant with incomplete penetrance	Sporadic	Likely loss-of-function mutations: Missense Insertions	Mild neurodevelopmental disorders Median age at thelarche: 5.4 (IQR 6.2) years

Abbreviation: CPP, central precocious puberty.

^*a*^To date, only 2 patients with isolated CPP were reported carrying activating mutations affecting the kisspeptin system—1 in the gene encoding kisspeptin (*KISS1*) and 1 in the gene encoding its receptor (*KISS1R*)—indicating that activating mutations in these genes are extremely rare (references 7 and 8).

## Basic Principles of Genetics and Epigenetics

Genetics is the study of heritable changes in gene activity or function resulting from direct alteration of the DNA sequence [15]. Genetic defects comprise point mutations, deletions, insertions, and translocations, involving a single gene (monogenic), multiple genes, or chromosomes. The genetic etiology of a clinical condition can be suspected by clinical aspects such as recurrence in the family, presentation early in life, and phenotypes involving multiple systems [16, 17]. Regarding monogenic conditions, the investigation can be based on single-gene tests (such as Sanger sequencing), where a particular candidate gene is studied according to the clinical presentation of the patient. However, in an unprecedented way, the genetic bases of numerous monogenic conditions have been revealed by the growing use of unbiased techniques with simultaneous sequencing of multiple genes (such as whole-exome or whole-genome sequencing) [17].

Epigenetics is the study of changes in gene activity or function that are not associated with changes in the DNA sequence itself [15, 18]. Epigenetic modifications are chemical additions to DNA and histones that play a key role in the regulation of chromatin configuration and gene expression patterns, ultimately leading to gene silencing or activation [19]. Therefore, epigenetic processes may alter phenotype without changing genotype, being a potential source of phenotypical variability [20]. To date, 3 main epigenetic regulatory mechanisms have been described in human biology: DNA methylation; posttranslational modifications of histone proteins; and regulation by noncoding RNAs (principally small noncoding RNAs named microRNAs) [11, 19]. Notably, the loss of effectiveness of epigenetic regulation has been associated with distinct human diseases, which may be caused by direct defects disrupting epigenetic mechanisms or by genetic mutations in genes encoding epigenetic regulators [20].

The best-known epigenetic mechanism is DNA methylation, a biological process by which methyl groups are added to DNA to repress or activate genes. Briefly, DNA methylation involves the transfer of methyl groups to the C5 position of cytosines to form 5-methylcytosines [15, 20]. In mammals, the principal targets of DNA methylation are the cytosines that precede a guanine, forming CpG dinucleotides [20]. DNA methylation regulates gene expression by recruiting proteins involved in gene repression or by inhibiting the binding of transcription factors to DNA [15]. DNA methylation machinery involves 3 classes of proteins: writers, which add the methyl groups onto cytosines; erasers, which remove the methyl group; and readers, which recognize and bind to methyl groups to ultimately influence gene expression [15, 21]. Notably, DNA methylation plays crucial roles in numerous biological processes, including genomic imprinting, X chromosome inactivation, regulation of tissue-specific gene expression and transposon silencing [21].

## The Role of Imprinted Genes

### Genomic Imprinting

Genomic imprinting is a physiological epigenetic process of gene silencing established through DNA methylation of promoter or regulatory regions by which selected genes are expressed according to its parental origin, leading to monoallelic expression [22, 23]. Genomic imprinting is under the control of differentially methylated regions know as imprinting control regions. In humans, approximately 200 genes distributed in clusters throughout 40 genomic regions were predicted to be imprinted, encompassing less than 1% of all genes [24, 25]. Imprinted genes play pivotal roles in development, growth, metabolism, and behavior [23, 25]. In addition, recent evidence indicated a relevant role for genomic imprinting in the central nervous system because numerous imprinted genes show imprinted expression in the brain [24]. Alterations affecting the dosage or expression of imprinted genes have been implicated in the pathogenesis of a group of human congenital syndromes known as imprinting disorders. The main molecular mechanisms underlying imprinting disorders are copy number variants, uniparental disomy, epimutation, or mutation of the expressed copy.

In the past decade, the identification of disease-causing inactivating mutations in 2 imprinted genes in multiple families with CPP has evidenced a strong role for genomic imprinting, as well as for epigenetics, in the control of puberty [3]. The first description of CPP-causing mutations in an imprinted gene was performed in a seminal study in 2013. Abreu, Dauber et al [26]. identified loss-of-function mutations in the imprinted gene Makorin Ring-Finger Protein 3 (*MKRN3*), which since then have been described as the most common cause of monogenic CPP. Subsequently, loss-of-function mutations in the imprinted gene Delta-Like Non-Canonical Notch Ligand 1 (*DLK1*) were described as a rarer cause of monogenic CPP [27]. Remarkably, *MKRN3* and *DLK1* are maternally imprinted genes, that is, both genes have the maternal allele silenced and only the paternal allele expressed. Therefore, in individuals carrying inactivating mutations in *MKRN3* or *DLK1*, CPP will develop if the mutation is on the paternal allele; conversely, CPP will not develop if the mutation is on the maternal allele [3, 26, 27].

Remarkably, 2 powerful large-scale GWAS demonstrated that, when paternally inherited, single nucleotide polymorphisms in *MKRN3* and *DLK1* can largely influence the age at menarche in women, a milestone in female pubertal development, reinforcing the role of both genes on pubertal timing [28, 29]. Additionally, another curious aspect is that *MKRN3* and *DLK1* are located at regions critical for imprinting disorders related with CPP at some degree. *MKRN3* is at the boundary of the region of Prader-Willi syndrome (PWS), which is associated with CPP in about 4% of cases, whereas *DLK1* is at the locus of Temple syndrome, a rare disorder characterized by CPP in 80% to 90% of cases [16]. Despite this, CPP phenotype resulting from mutations in *MKRN3* or *DLK1* have been characterized as nonsyndromic forms of CPP, as outlined in the sections that follow.

Evolutionary studies have considered that genomic imprinting has evolved in mammal species according to the kinship theory, where imprinting is the inevitable consequence of conflictive selective forces acting on differentially expressed parental alleles [30]. The identification of inactivating mutations in the imprinted genes *MKRN3* and *DLK1* in children with familial CPP has contributed to the evolutionary premise regarding puberty. Kotler and Haig [31] have suggested that imprinted genes could influence events of pubertal development, with paternally expressed genes promoting delay in maturation and maternally expressed genes promoting acceleration in maturation. In this theory, the tempo of human childhood development is believed to have a maternal foot on the accelerator and a paternal foot on the brake [31].

### MKRN3—A Brake on GnRH Secretion During Childhood

The *MKRN3* was first linked to the reproductive axis in 2013 with the identification of gene mutations in families with CPP using whole-exome sequencing [26]. In this report, 4 deleterious *MKRN3* mutations—3 frameshift and 1 missense—were detected in individuals with familial CPP from both sexes from 5 of 15 families (33%). After this, loss-of-function mutations in *MKRN3* were also identified in patients with CPP but without a clear family history [32]. Since then, several studies from different countries have described distinct loss-of-function mutations in *MKRN3* in patients with CPP [33]. *MKRN3* mutations are now the most common genetic defect associated with CPP, with an overall frequency around 9% among idiopathic cases of CPP; this frequency is significantly higher in familial cases of CPP, ranging from 22% to 46% [34-36].

Human *MKRN3* is located on the chromosome 15q11.2 in a region with a cluster of imprinted genes that can be paternally or maternally expressed [37, 38]. The disruption of expression of paternally expressed genes from chromosome 15q11.2 is associated with PWS. In this complex syndrome, both delayed and precocious pubertal development have been described. Notably, hypogonadism is the most common reproductive manifestation, being considered a prevalent clinical feature of the syndrome [39]. Studies have shown that *MKRN3* deletion is neither required nor responsible for the PWS phenotype, with several cases of PWS presenting without deletion of *MKRN3* [39, 40]. Additionally, the inclusion of *MKRN3* within the deletion causing PWS did not predict the pubertal phenotype, as most patients with PWS with deletions including *MKRN3* did not develop CPP [41, 42]. To date, few cases were described with PWS and CPP, which is likely due to the deletion of the paternal copy of *MKRN3* [39, 43, 44]. Indeed, Meader et al [42]. suggested a range of pubertal developmental trajectories among patients with distinct 15q11.2 deletions including *MKRN3*. In this study, it was recognized that patients with larger 15q11.2 deletions, including most factors of the PWS critical region, had PWS phenotype without early pubertal development, whereas those with smaller deletions, including only *MKRN3* or *MKRN3* and additional few factors next to it, did not have PWS phenotype, but had early pubertal development. These findings suggested that the CPP phenotype might not be reported in most patients with PWS because the concomitant deletion of factors on chromosome 15q11.2 related to hypogonadism likely overcome the loss of *MKNR3,* leading to an inability to secrete early pubertal levels of gonadotropins or sex steroids [3].

**Figure 2.**
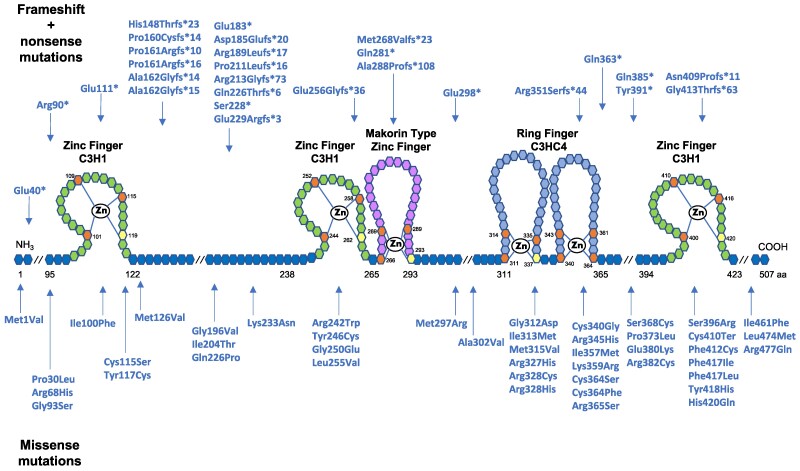
Schematic representation of MKRN3 protein structure and 73 mutations identified in patients with central precocious puberty. Hexagons represent individual amino acids; corresponding numbers indicate amino acid position. Top row mutations are frameshift and nonsense, whereas the bottom row are missense mutations. The functional domains of MKRN3 protein contain Key cysteine and histidine amino acids, which are necessary for zinc ion interaction. RING finger C3HC4 is a protein-binding domain responsible for ubiquitin ligase activity. Zinc-Finger C3H1 are RNA-binding domains. Makorin type zinc-finger is a specific Cys-His domain identified in the proteins of the makorin family. Notably, 19 mutations were detected between the first 2 C3H1 domains, 14 of which are frameshift. Mutations tend to also cluster within the C3HC4 RING finger domain, the vast majority of which are missense.

To date, more than 70 inactivating mutations in the coding region of *MKRN3* have been described, including missense (63%), frameshift (27%), and nonsense mutations (10%) [3]. Figure 2 shows an overview of mutations in the coding region of *MKRN3* identified so far. The most prevalent *MKRN3* mutation is an indel variant affecting a poly-C region (cDNA positions 475 to 481) that results in a frameshift mutation with a premature stop codon with an amino-acid change either at Pro160, Pro161, or Ala162 [33]. Notably, 2 unrelated American girls with nonsyndromic CPP were found to have heterozygous paternally inherited whole-gene deletions of *MKRN3* [42]. In addition, rare cases of paternally inherited mutations in the promoter region of *MKRN3* have also been identified in patients with CPP [45].

Notably, most studies demonstrated no significant differences in clinical and laboratory features of patients with CPP resulting from *MKRN3* mutations in comparison with patients with idiopathic CPP [32, 33]. A large multiethnic cohort of 71 patients with CPP resulting from *MKRN3* mutations identified mean age at first pubertal signs of 6.2 ± 1.2 years in girls and 7.1 ± 1.5 years in boys [33]. It has been proposed that boys with loss-of-function mutations in *MKRN3* have a smaller advance in the timing of puberty onset compared to girls [46]. However, this might reflect an underestimate in the diagnosis of male CPP because of the difficulties in identifying testicular enlargement, the first clinical evident sign of pubertal onset in boys. Despite that, a cohort study identified loss-of-function mutations in *MKRN3* in 5 of 20 (40%) boys with apparently idiopathic CPP, demonstrating a high frequency of this monogenic condition in the male sex [47].

MKRN3 is a member of the Makorin protein family that is highly conserved among species [37, 38]. The MKRN3 protein has a centrally located RING finger motif (C3HC4), 2 amino-terminal C3H zinc-finger motifs followed by a Makorin zinc-finger motif unique to the Makorin protein family, and a carboxy-terminal C3H zinc-finger motif [38]. These multiple domains suggest that MKRN3 has multiple actions. RING zinc-finger motif is responsible for E3 ubiquitin ligase activity, whereas C3H zinc-finger motifs have been implicated in mRNA binding [48]. Additionally, a functional significance for the *MKRN3* 3′UTR has long been suggested because of its conserved nature [38]. Indeed, experimental studies demonstrated that the *MKRN3* 3′UTR might be a binding site for miR-30, a repressor microRNA. Functional in vitro analyses demonstrated a strong repressive action of miR-30 on *MKRN3* 3′UTR and consequent reduction of MKRN3 expression [49]. Additionally, circulating relative miR-30b levels were assessed in boys with delayed puberty, showing an increase during puberty and suggesting a relation with the human HPG axis activity [50]. Indeed, miR-30 was the first element shown to regulate *MKRN3* expression. Changes in miR30 and other microRNAs causing CPP in humans are still to be unmasked [3].

Compelling evidence has shown that *MKRN3* acts as a brake on GnRH secretion during childhood. Experimental studies have demonstrated potential mechanisms of *MKRN3* action, including ubiquitination, autoubiquitination, alterations of GnRH expression, and interactions with transcription factors [51]. *MKRN3* is an E3 ubiquitin ligase that undergoes autoubiquitination. Ubiquitination is a process of regulated degradation of cell proteins. Li et al [52]. demonstrated that *MKRN3* regulated *GNRH1* (GnRH gene) transcription through ubiquitination of Methyl-CpG-binding Domain Protein 3 (MBD3), disrupting its binding to the *GNRH1* promoter and thus epigenetically silencing the *GNRH1* transcription. MBD3 is a component of the Methyl-CpG-binding domain (MBD) family. Additionally, a subsequent study identified that ubiquitination of Poly(A)-binding proteins by MRKN3 lead to shortening of the Poly(A) tail length of *GNRH1* mRNA, compromising the formation of its translation initiation complex [53]. These data indicated that MKRN3 can control both transcriptional and posttranscriptional switches of pubertal initiation.

Proteomic studies showed that MKRN3 interacted with IGF 2 mRNA-binding Protein 1 and Poly(A)-binding Protein Cytoplasmic 1, indicating a role of MKRN3 on mRNA metabolism [54]. Also, a study in mice indicated that Mkrn3 suppressed the activity of Nptx1, an important protein for neuron development [55]. The RING finger domain of Mkrn3 was shown to be essential for binding with Nptx1 for its polyubiquitination during puberty initiation. Notably, 2 distinct studies evaluated the functional impact of 4 missense *MKRN3* mutations previously identified in patients with CPP; 2 (p.Cys340Gly and p.Arg365Ser) were located on the RING finger domain and 2 (p.Phe417Ile and p.His420Gln) were located on the C-terminal zinc-finger domain [52, 56]. Abreu et al [56] identified that the *MKRN3* mutations affecting the RING finger domain comprised the ability of MKRN3 to inhibit *KISS1* and *TAC3* promoter activity. Additionally, Li et al [52] demonstrated that the 4 *MKRN3* mutations resulted in loss of the ability to inhibit *GNRH1* promoter activity. These data confirmed that these mutations resulted in loss-of-function of MKRN3, while also implicated the RING finger ubiquitin ligase domain as an important functional domain of this inhibitory factor of puberty.

Studies in mice identified a high *MKRN3* expression in the hypothalamus before sexual maturation that declined with pubertal development [26]. The same pattern was shown in rats and in female nonhuman primates [56]. Importantly, in rodents, hypothalamic expression of *MKRN3* was shown to be more specific to KNDy neurons. The decline of *MKRN3* expression before sexual maturation in mice was independent of changes in sex steroids and leptin, indicating that *MKRN3* regulation might be upstream in the HPG axis [56, 57]. Indeed, Abreu et al [56] demonstrated in vitro that MKRN3 inhibited the transcription of human *KISS1* and *TAC3*, 2 key stimulators of GnRH secretion.

The identification of loss-of-function mutations in *MKRN3* in humans strongly implicated this gene with puberty onset. As discussed here, since we linked *MKRN3* with the age of pubertal initiation, several human, animal, and in vitro data showed that *MKRN3* is a component of the hypothalamic inhibitory input on GnRH neurons through different mechanisms.

### DLK1—A Link Between Reproduction and Metabolism

In 2017, Dauber et al [27] described the first association of *DLK1* deficiency with monogenic CPP. An intragenic deletion of *DLK1* was identified in a large multigenerational Brazilian family with nonsyndromic CPP through an innovative approach, including linkage analysis followed by whole-genome sequencing. The *DLK1* mutation followed a pattern of autosomal dominant inheritance with paternal transmission, as expected for a maternally imprinted gene. Later, 3 distinct frameshift mutations in *DLK1* were described in females with CPP or precocious menarche from 3 unrelated families from Brazil and the United Kingdom, whereas a de novo splice site deletion in *DLK1* was identified in a Spanish girl with sporadic CPP [58, 59]. Notably, further studies identified frameshift mutations in *DLK1* in 2 boys with familial CPP from different ethnic backgrounds [60, 61]. More recently, novel inactivating mutations in *DLK1* were reported in 2 unrelated French girls and in 2 unrelated Turkish girls with nonsyndromic familial CPP [62, 63]. Therefore, to date, a total of 10 distinct mutations in *DLK1* have been reported in 28 patients with CPP from 11 unrelated families from 7 countries. Despite it being rare, current evidence shows that loss-of-function mutations in *DLK1* are a definitive cause of monogenic CPP. Notably, affected individuals had a higher frequency of adverse metabolic outcomes in adulthood, including principally overweight/obesity, hyperlipidemia, and early-onset glucose intolerance/type 2 diabetes mellitus, highlighting a potential role for *DLK1* in the intersection between reproductive function and metabolic processes [58, 60].

Human *DLK1* gene is a paternally expressed gene located at *DLK1-DIO3* locus on the chromosome 14q32.2, a complex region that contains several imprinted genes involved in critical biological processes such as development, behavior, and metabolism [64]. The disruption of the methylation pattern of the imprinted 14q32.2 region is associated with Temple syndrome, a rare imprinting disorder marked by CPP, as well as pre- and postnatal growth failure, small hands/feet, motor/speech delay, obesity, and early-onset type 2 diabetes [65, 66]. Current evidence indicates that DLK1 deficiency is the leading mechanism of CPP in this condition [16].

*DLK1*, also referred as Preadipocyte factor 1, encodes a transmembrane protein containing 6 epidermal growth factor-like motifs in its extracellular domain [67]. It is a noncanonical ligand of the Notch signaling pathway, functioning in a competitive inhibitory manner [68]. The DLK1 intracellular domain has been shown to be a negative regulator of Notch signaling by disrupting the Notch1/RBP-JK transcriptional complex [27]. The Notch pathway is essential for regulating cell differentiation, tissue homeostasis, and cell fate [67]. Classically, *DLK1* is known to function as a negative regulator of adipogenesis, by preventing the differentiation of preadipocytes into mature adipocytes [68]. In humans, DLK1 is widely expressed in fetal tissues, but postnatal expression is restricted to endocrine tissues, such as adrenals, pituitary, and ovaries [27]. Additionally, DLK1 was shown to be expressed in several hypothalamic nuclei, including the ARC and the AVPV nuclei, at postnatal life [69]. In animal models, *Dlk1*-deficient mice reached puberty at significantly lower body weights, suggesting that the absence of DLK1 might attenuate the impact of reduced body weight on pubertal timing in these animals [70]. Additionally, *Dlk1*-deficient mice exhibited accelerated adiposity and hyperlipidemia in adulthood [71].

Because of alternative splicing, there are different forms of DLK1 protein, mainly soluble and membrane-bound isoforms. The soluble form of DLK1 is biologically active and can be generated by mediated cleavage of its extracellular domain, making DLK1 a measurable serum protein [72]. To investigate the effect of mutations on DLK1 production, serum DLK1 levels have been measured using an available soluble DLK1 ELISA [27]. To date, all *DLK1* mutations described in patients with CPP have resulted in undetectable or very low serum DLK1 levels compared to control individuals [3]. These data indicate that serum DLK1 measurement might be a screening method for investigating DLK1 deficiency in affected individuals.

Although all of these observations reinforce the potential connection between DLK1 biology, reproduction, and metabolism, the exact mechanism by which *DLK1* regulates pubertal timing remains to be fully elucidated. Current evidence suggested that *DLK1* plays a critical role in the differentiation and function of hypothalamic neurons that regulate the HPG axis, particularly kisspeptin neurons in the ARC and AVPV nuclei. Indeed, in hypothalamus, an active Rbp-jk-mediated Notch signaling was identified as critical for instructing subpopulations of progenitor cells to differentiate into kisspeptin neurons [73]. This implied that *DLK1* might impact hypothalamic neurogenesis and influence formation, maturation, and secretion of kisspeptin neurons by modulating Notch signaling pathways during embryogenesis and throughout postnatal life [73].

## The Putative Role of an X-linked Gene

### X Chromosome Inactivation and X-linked Disorders

X chromosome inactivation is a normal mechanism of gene silencing mediated by methylation occurring in females, characterized by the silencing of 1 X chromosome in every cell to ensure dosage compensation, equalizing the expression of X-linked genes between both sexes [74-76]. This process is random and has tissue-specific patterns, making women mosaic, for which the X chromosome is active (paternal or maternal). X-linked genes may have dominant or recessive patterns of inheritance [76]. Males are in a hemizygous state, being expected to be affected when carrying a X-linked mutation. Females carrying heterozygous mutations in dominant X-linked genes can be affected, mildly affected, or unaffected. This variable phenotypic expressivity can occur because of tissue-specific patterns of X chromosome inactivation [75, 76].

X-linked gene defects have long been considered likely causative of central nervous system disorders because these genes are highly expressed in the brain [74, 77]. The potential role of X-linked genes in human pubertal development has been suggested by distinct lines of evidence, including reports of early puberty in individuals with X-chromosome structural variants, enrichment of X-linked differentially methylated regions in methylome profiling of girls at puberty, and GWAS in large populations [78-80].

### Reproduction and Neurodevelopment—new Perspectives

Promising data have suggested that there seem to be an overlap between the network of factors controlling puberty and reproduction and the network of factors controlling neurodevelopment and cognition. Experimental studies indicated the involvement of GnRH neurons in the control of postnatal brain development and adult cognition [81]. A very elegant pilot study demonstrated that GnRH replacement rescued cognition in Down syndrome in mice models and human adult patients [82]. Furthermore, GnRH-dependent pubertal disorders, such as precocious or delayed puberty, have long been reported among the phenotypes of distinct neurodevelopmental syndromes caused by genetic and/or epigenetic defects [3, 6].

Notably, this overlap has been also shown by population studies. Recently, 2 distinct large cohort studies, 1 in the United States and another in Taiwan, investigated the risk of presenting precocious puberty among children with autism spectrum disorder [83, 84]. Both studies identified that children with autism had an increased risk of precocious puberty, adding evidence to the possibility of common mechanisms between both conditions.

### MECP2—A Potential Common Factor Between Puberty and Neurodevelopment

Recently, an additional monogenic factor was associated with CPP. Rare heterozygous variants in the Methyl-CpG-Binding Protein 2 (*MECP2*) gene, an epigenetic factor, were identified in multiple girls with CPP with or without neurodevelopmental disorders from a large multiethnic cohort [77]. Human *MECP2* is an X-linked dominant gene located on chromosome Xq28 and subject to X inactivation. It encodes a methylated DNA nuclear protein that functions as repressor or activator of gene transcription [85]. In addition, recent studies in animal models have identified that MECP2 is involved in gene repression and activation through binding to non-CG methylation and recruitment of co-repressor and coactivator complexes, highlighting that neuronal non-CG methylation is also a target for MECP2 function [86, 87]. MECP2 is a reader protein from the DNA methylation machinery, being a component of the MBD family (same family as the MBD3 protein, associated with MKRN3 function), a group of proteins that functions as transcription factors of DNA methylation [21]. Human *MECP2* consists of transcription start site, 4 exons, 3 introns, and multiple polyadenylation sites. Because of alternative splicing, there are 2 protein isoforms, MeCP2E1 and MeCP2E2; MeCP2E1 has higher expression in the brain. The main functional domains of MECP2 are amino-terminal, methyl-binding, intervening, transcriptional repression, and carboxy-terminal domains [88].

*MECP2* has a well-established role in neurodevelopment, mainly in neuronal maturation and remodeling. Loss-of-function mutations in *MECP2* are usually associated with neurodevelopmental disorders, particularly with Rett syndrome [89]. This neurodevelopmental syndrome usually is a severe disorder characterized by the regression of acquired skills between age 12 and 30 months old. Its main diagnostic criteria are loss of acquired purposeful hand skills and spoken language, abnormal gait, and stereotypic movements principally from the hands [90]. Rett syndrome is a dominant X-linked disorder that has a frequency markedly higher in females than in males. Because of the advent of multigene sequencing approaches, *MECP2* mutations have also been identified in patients with milder neurodevelopmental disorders, such as autism or intellectual deficiency. Notably, early pubertal development has been demonstrated in children with Rett syndrome resulting from *MECP2* mutations, such as precocious puberty in 15% and early thelarche in 25% of girls [91-93].

In a recent study, Canton et al [77] used multigene sequencing in 133 patients and single-gene sequencing in additional 271 patients, investigating a total of 404 patients with idiopathic CPP from 5 different countries. Rare potentially damaging heterozygous variants in *MECP2* were identified in 7 girls with sporadic CPP. Variants were de novo in 4 girls, reinforcing the evidence of pathogenicity of such genetic defects. Clinical heterogeneity was identified probably because of the severity of variants, where the more damaging the variant, the more complex the phenotype. Three girls with de novo missense mutations had CPP associated with mild neurodevelopmental disorders, such as microcephaly or autism spectrum disorder, probably representing part of the spectrum of neurodevelopmental disorders related to *MECP2*. Four girls had insertion variants and did not manifest clear neurodevelopmental findings, suggesting milder clinical pictures. Importantly, no patient had Rett syndrome.

Remarkably, the evaluation of hypothalamic expression of Mepc2 in female mice demonstrated that Mecp2 protein colocalized with GnRH expression in hypothalamic nuclei responsible for GnRH regulation [77]. In addition, the study of hypothalamic expression of MECP2 in ewes identified that mRNA expression levels of MECP2 decreased, whereas GnRH and KISS1 increased significantly during puberty onset [94]. This evaluation in ewes also showed a hypothalamic coexpression of MECP2 protein with GnRH and kisspeptin. The exact mechanism of action of *MECP2* in the reproductive axis is still to be elucidated. Nevertheless, the identification of rare variants in *MECP2* in multiple girls with idiopathic CPP with or without mild neurodevelopmental disorders suggested a potential X-linked form of premature pubertal development.

## Conclusions

The discovery of genes associated with CPP has contributed to the current concept that genetics and epigenetics are in the core of human pubertal timing, reinforcing findings from animal models and experimental studies. Loss-of-function mutations in *MKRN3*, *DLK1,* and *MECP2* have been associated with CPP. Notably, the expression of *MKRN3* and *DLK1* genes is regulated by genomic imprinting, an epigenetic mechanism mediated by DNA methylation, whereas *MECP2* gene has a potential role as an epigenetic regulator of gene transcription because it functions as a key element of the DNA methylation machinery. Indeed, these discoveries have expanded the etiology of CPP. A recent analysis of a large cohort of patients with CPP identified an overall frequency of genetic etiologies of 12.6% (22.2% in boys and 12.1% in girls) among patients with apparently idiopathic CPP, demonstrating that the genetic etiology is a relevant cause of CPP in both sexes [9]. This scenario might allow patients with CPP to benefit from a precision medicine approach, providing them more personalized strategies of follow-up with increased long-term clinical surveillance [77]. In addition, genetic and epigenetic discoveries underlying CPP might contribute to the development of potential future treatment targets in the field of human reproduction.

## Data Availability

This is a narrative mini-review based on published data. Accordingly, specific data sharing is not applicable to this article as no new datasets were generated or analyzed during the current study.

## Abbreviations

ARC: arcuate nucleus
AVPV: anteroventral periventricular nucleus
CPP: central precocious puberty
GWAS: genomewide association studies
HPG: hypothalamic-pituitary-gonadal
MBD: Methyl-CpG-binding domain
PWS: Prader-Willi syndrome
